# Practical quality improvement changes for a low-resourced pediatric unit

**DOI:** 10.3389/fpubh.2024.1411681

**Published:** 2024-06-12

**Authors:** Phoebe H. Yager, Kevin Mary Callans, Aubrey Samost-Williams, Jose A. Bonilla, Luis J. G. Flores, Susana C. A. Hasbun, Angel E. A. Rodríguez, Alejandra B. A. Cárdenas, Alexia M. L. Núñez, Asitha D. L. Jayawardena, Evelyn J. Zablah, Christopher J. Hartnick

**Affiliations:** ^1^Massachusetts General Hospital, Boston, MA, United States; ^2^Department of Anesthesiology, McGovern Medical School at the University of Texas Health Science Center at Houston, Houston, TX, United States; ^3^Department of Pediatric Otolaryngology, Hospital de Niños y Adolescentes Centro Pediatrico, San Salvador, El Salvador; ^4^Department of Pediatric Critical Care Medicine, Hospital Centro Pediátrico, San Salvador, El Salvador; ^5^Department of Anesthesiology, Hospital Centro Pediátrico, San Salvador, El Salvador; ^6^Department of Pediatrics, Hospital Centro Pediátrico, San Salvador, El Salvador; ^7^Instituto Tecnológico y de Estudios Superiores de Monterrey in Guadalajara, Zapopan, Mexico; ^8^Children’s Minnesota, ENT & Facial Plastic Clinic, Minneapolis, MN, United States; ^9^Department of Otolaryngology, Massachusetts Eye and Ear Infirmary, Boston, MA, United States

**Keywords:** quality improvement, pediatric intensive care unit, low/middle income country, unplanned extubations, Mass General Brigham, institutional review

## Abstract

**Background:**

This work describes a sustainable and replicable initiative to optimize multi-disciplinary care and uptake of clinical best practices for patients in a pediatric intensive care unit in Low/Middle Income Countries and to understand the various factors that may play a role in the reduction in child mortality seen after implementation of the Quality Improvement Initiative.

**Methods:**

This was a longitudinal assessment of a quality improvement program with the primary outcome of intubated pediatric patient mortality. The program was assessed 36 months following implementation of the quality improvement intervention using a *t*-test with linear regression to control for co-variates. An Impact Pathway model was developed to describe potential pathways for improvement, and context was added with an exploratory analysis of adoption of the intervention and locally initiated interventions.

**Results:**

147 patients were included in the sustainability cohort. Comparing the initial post-implementation cohort to the sustainability cohort, the overall PICU unexpected extubations per 100 days mechanical ventilation decreased significantly from baseline (6.98) to the first year post intervention (3.52; *p* < 0.008) but plateaued without further significant decrease in the final cohort (3.0; *p* = 0.73), whereas the mortality decreased from 22.4 (std 0.42) to 9.5% (std 0.29): *p* value: 0.002 (confidence intervals: 0.05;0.21). The regression model that examined age, sex, diagnosis and severity of illness (via aggregate Pediatric Risk of Mortality (PRISM) scores between epochs) yielded an adjusted R-squared (adjusting for the number of predictors) value of 0.046, indicating that approximately 4.6% of the variance in mortality was explained by the predictors included in the model. The overall significance of the regression model was supported by an F-statistic of 3.198 (*p* = 0.00828). age, weight, diagnosis, and severity of illness. 15 new and locally driven quality practices were observed in the PICU compared to the initial post-implementation time period. The Impact Pathway model suggested multiple unique potential pathways connecting the improved patient outcomes with the intervention components.

**Conclusion:**

Sustained improvements were seen in the care of intubated pediatric patients. While some of this improvement may be attributable to the intervention, it appears likely that the change is multifactorial, as evidenced by a significant number of new quality improvement projects initiated by the local clinical team. Although currently limited by available data, the use of Driver Diagram and Impact Pathway models demonstrates several proposed causal pathways and holds potential for further elucidating the complex dynamics underlying such improvements.

## Introduction

The United Nations’ Sustainable Development Goal is to reduce mortality under five years of age to fewer than 25 lives per 1,000 (2.5%) by 2030 (1). Settings characterized by elevated mortality rates within low and middle-income countries (LMIC) represent optimal targets for intervention, as the relatively high number of cases presents the most substantial opportunity for impact. One such setting is pediatric intensive care units (PICUs) in these countries, which often see mortality rates ten times higher than those in high-income countries (25–50% vs. 2–5%) (2–5). Intubated children are at particularly high risk. Data collected from one PICU in a LMIC Latin American country, found all-cause PICU mortality in intubated patients to be 24%, with an unplanned extubation (UE) rate of 31%, compared to a rate of approximately 2% in the United States (6). Unplanned extubation involves the unplanned and unintentional dislodgement of an endotracheal tube and contributes to significant morbidity, including hypoventilation, hypoxia, ventilator-associated pneumonia, prolonged mechanical ventilation, acquired subglottic stenosis, hemodynamic collapse, and death (7, 8).

Operation Airway is a surgical mission-based nonprofit organization of Massachusetts Eye and Ear (MEE) and is composed of pediatric providers from MEE and Massachusetts General Hospital, both teaching hospitals of Harvard Medical School. Careways Collaborative was born from Operation Airway and is a nonprofit organization that designs, disseminates, and studies educational programs and QI interventions aimed at reducing pediatric mortality in LMICs. In 2017, Careways Collaborative partnered with Hospital Nacional de Niños Benjamín Bloom, the largest public children’s hospital in El Salvador, to develop and deploy a QI protocol, wherein a multidisciplinary, video-based educational curriculum is provided to PICU staff with a goal of optimizing safe care of intubated patient and thereby reducing PICU mortality.

To evaluate and iteratively improve the QI protocol, we collected pre-intervention data for 18 months (Epoch 1), then implemented the initial educational intervention and tracked PICU airway-related morbidity and mortality for three months (Epoch 2). We observed a significant decrease in unplanned extubations/100 days Mechanical Ventilation (UE/MV) (6.98 to 3.52) and pediatric intensive care unit (PICU) mortality (26·7 to 22·4%) between Epoch 1 and 2 (9). Following Epoch 2, there was a 36-month period with no in-person mission trips from Careways due to the COVID-19 pandemic. When mission trips resumed in January 2023, we had an opportunity to assess longer-term impacts of the QI program. This Quality Report describes findings from Epoch 3, including how local health teams adapted the QI protocols over time to meet local needs and reports on the sustained impacts of the protocol.

## Materials and methods

### Intervention design and background

Hospital Nacional de Niños Benjamín Bloom is the largest public children’s hospital in El Salvador. It is the only academic, stand-alone children’s hospital in El Salvador and it cares for some of the most disadvantaged children in the country and patients with the most complex medical needs. It is a mixed medical surgical unit with 16 beds, staffed during the day by a pediatric resident and PICU fellow with a pediatric intensivist on site part-time; there is one on-site respiratory therapist at all times; and the nursing ratio is 1:4. Available monitoring includes pulse oximetry, respiratory rate, non-invasive blood pressure, telemetry, central venous pressure, and arterial pressure. Available supportive technologies include hemodialysis and mechanical ventilation.

During a mission trip to Hospital Nacional de Niños Benjamín Bloom in 2017, Operation Airways (OA) and hospital staff developed a longitudinal database to count airway related morbidity and mortality in the PICU (10). In parallel, we identified knowledge gaps and communication failures possibly contributing to unplanned extubations and other adverse events. In response, we designed the initial educational intervention and QI protocols outlined below, aiming to: (1) enhance the knowledge of every member within the care team; (2) foster collaboration and improve communication among team members; and (3) cultivate a safety-oriented culture wherein team members approach each case systematically, ultimately minimizing preventable errors and expediting issue identification.

**Figure 1:**
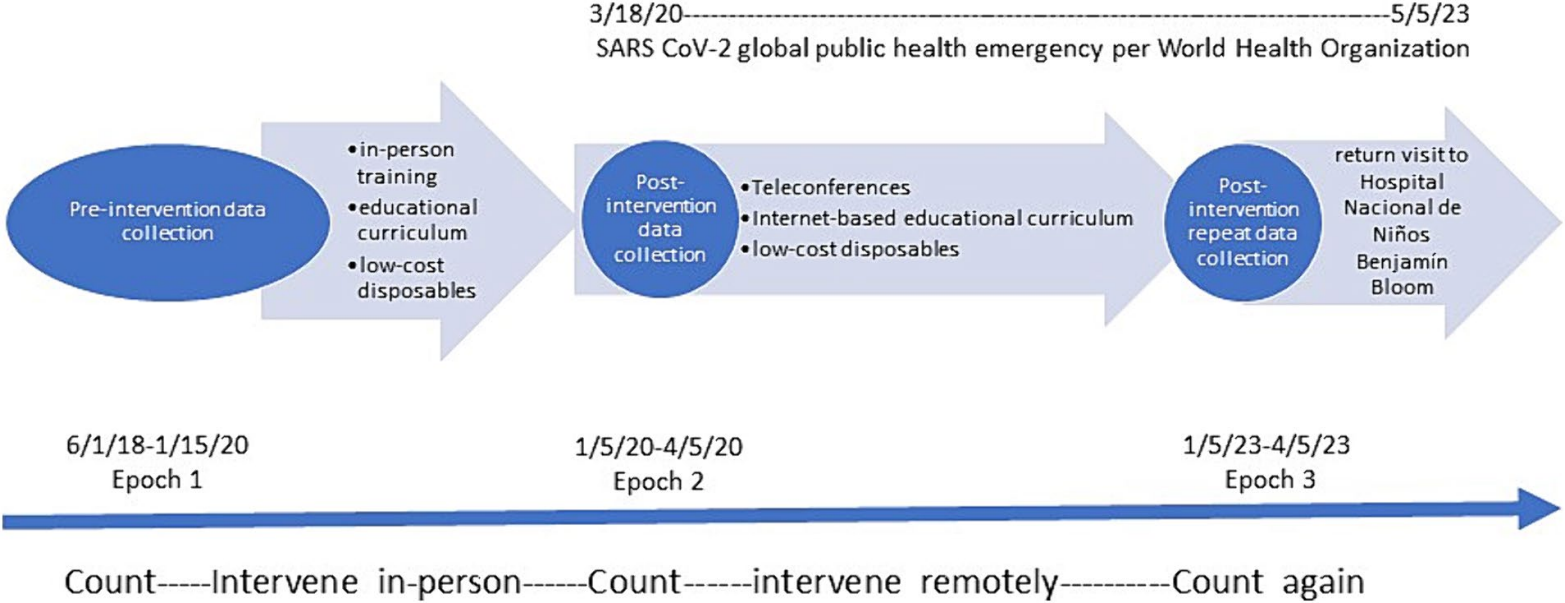
Timeline of pre-post intervention study. Timepoint 0 indicates start of 18-month pre-intervention period of data collection. Timepoint 1 indicates implementation of multi-media, in-person education-based intervention. Timepoint 2 when short-term post-intervention data collection period occurred. Timepoint 3 indicates 18-month period when no research staff visited the Bloom Hospital and relied on light-touch, internet-based support of the Bloom Hospital PICU team. Timepoint 4 indicates repeat post-intervention data collection during the same season as Timepoint 2. Timepoint 5 indicates return visit to the Bloom Hospital 18 months later.

The overall QI implementation and project timeline is shown in Figure 1. Epoch 1 began with 18 months of collecting pre-intervention data, after which we implemented the initial QI and educational intervention. Post-implementation data was then collected for three months (January through April) (Epoch 2) (9). Findings from this initial pre-post intervention study has been published elsewhere (9). Data, including demographics, illness severity scores, child age and weight, incidence of unexplained events, severity of illness (utilizing the Pediatric Risk of Mortality (PRISM) severity of illness instrument) (11–13), and mortality were recorded by a dedicated bilingual respiratory therapist with periodic audits by two independent reviewers to ensure accuracy (these two audits were performed by chart review by two local physicians who were not part of the primary research team). The study was not considered human subjects research by the guidelines of the Mass General Brigham (MGB) and Hospital Nacional de Niños Benjamín Bloom Institutional Review Board’s (IRB).

Following Epoch 2, there was a 36-month period with no data collection due to the COVID-19 pandemic. During this period, the local multidisciplinary surgical and PICU teams continued to have access to the internet-based educational curriculum, including a series of brief video-based tutorials and demonstrations covering topics such as how to safely turn the intubated patient, how to select appropriately sized ETTs, and how to monitor safe ETT cuff pressure. Once mission trips resumed, we resumed data collection on PICU morbidity and mortality data for 3 months, from January to April, 2023 (Epoch 3). The choice of the three months mirrored those months where data was collected for Epoch 2 and to account for any seasonal variation. Data collection in Epoch 3 was performed by the same dedicated bilingual respiratory therapist trained during the initial implementation, with audits performed by an independent reviewer for accuracy. The OA mission team also collected qualitative observational data on the presence or absence of the QI intervention components that had been previously implemented as well as any new QI interventions undertaken by the local team (Table 1). These local based QI interventions were developed by the local PICU team using the Quality Improvement framework they had learned previously specific but now expanded to include daily discussions and review of non airway factors such as dentition and nutrition, to reconfigure the PIVU space to allow for better ventilation and easier access for the nurses to provide care, to include routing periodic simulation as part of their team training.

**Table 1:** Observed pragmatic, practical changes made by the local health care team and observed in the Bloom Hospital PICU June 2022.

Element	Careways driven	Careways driven	Locally driven
	Education-based intervention	Observed Epoch 2	Observed Epoch 3
Implementation of daily ventilator associated pneumonia prevention bundle			
Head of bed elevated 30 degrees	✓	✓	✓
Daily oral hygiene			✓
Daily team discussion regarding extubation readiness	✓	✓	✓
Addition of a dentist to the PICU team to evaluate/treat patients with poor dentition			✓
Early initiation of enteral nutrition			✓
Consideration for early trachostomy placement in patients with expected lengthy need for invasive ventilation			✓
Increased use of cuffed endotracheal tubes (ETTs)	✓	✓	✓
Creation of a reference chart for proper sizing of cuffed ETTs	✓	✓	✓
Daily assessment of cuff leak to prevent over- or under-inflation	✓	✓	✓
Emergency card at patient’s head of bed including:			
Name			✓
Diagnosis			✓
Weight			✓
Lines			✓
ETT size			✓
Calculated code dose of epinephrine			✓
Multidisciplinary huddle at 6 AM with charge nurse, bedside nurse, respiratory therapist, intensivist and surgeon	✓	✓	✓
Formalization of pediatric critical care medicine fellowship			✓
Improved sedation with adoption of dexmedetomidine as mainstay of therapy			✓
Use of non-invasive ventilation to enable earlier extubation			✓
Increased use of standardized securement methods for orotracheal and nasotracheal ETTs	✓	✓	✓
Expansion of PICU floor plan allowing for more spacing between patient beds for easier access for nursing care.			✓
Initiation of an in-house simulation-based educational program for multidisciplinary PICU team	✓	✓	✓

A number of unanticipated practice changes not formally included in the education-based intervention were noted by the research team upon their return to the Bloom Hospital 18 months after their last visit. Some changes reflected formalization of practices modeled by the research team such as placement of an emergency card at the head of each ICU bed, while others were completely new initiatives like increasing the space between ICU beds to address infection control concerns.

### Intervention

**Figure 2:**
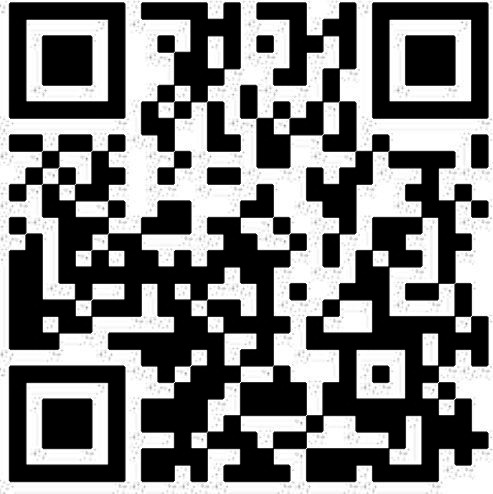
QR code for Spanish-Language Educational Video. Example of one of many QR codes posted in the PICU linking providers to 2–4 min Spanish-language instructional videos addressing safe airway management procedures and practices such as proper securement of the ETT, proper ETT selection, safe suctioning practices and safe management of cuff pressure in cuffed ETTs.

The intervention entails the implementation of the Careways QI protocol, which (1) provides multidisciplinary, video-based educational curriculum to PICU staff; (2) models multidisciplinary team rounding; (3) provides on-line readings and video modules accessible via QR codes posted across the PICU. The internet-based educational curriculum includes a series of brief video-based tutorials and demonstrations with new installations added over time covering topics such as how to safely turn the intubated patient, how to select the appropriately sized ETT, and how to monitor safe ETT cuff pressure. Figure 2 provides an example of a QR code that links to a Spanish-language video on how to properly secure an ETT.

### Driver diagram

**Figure 3:**
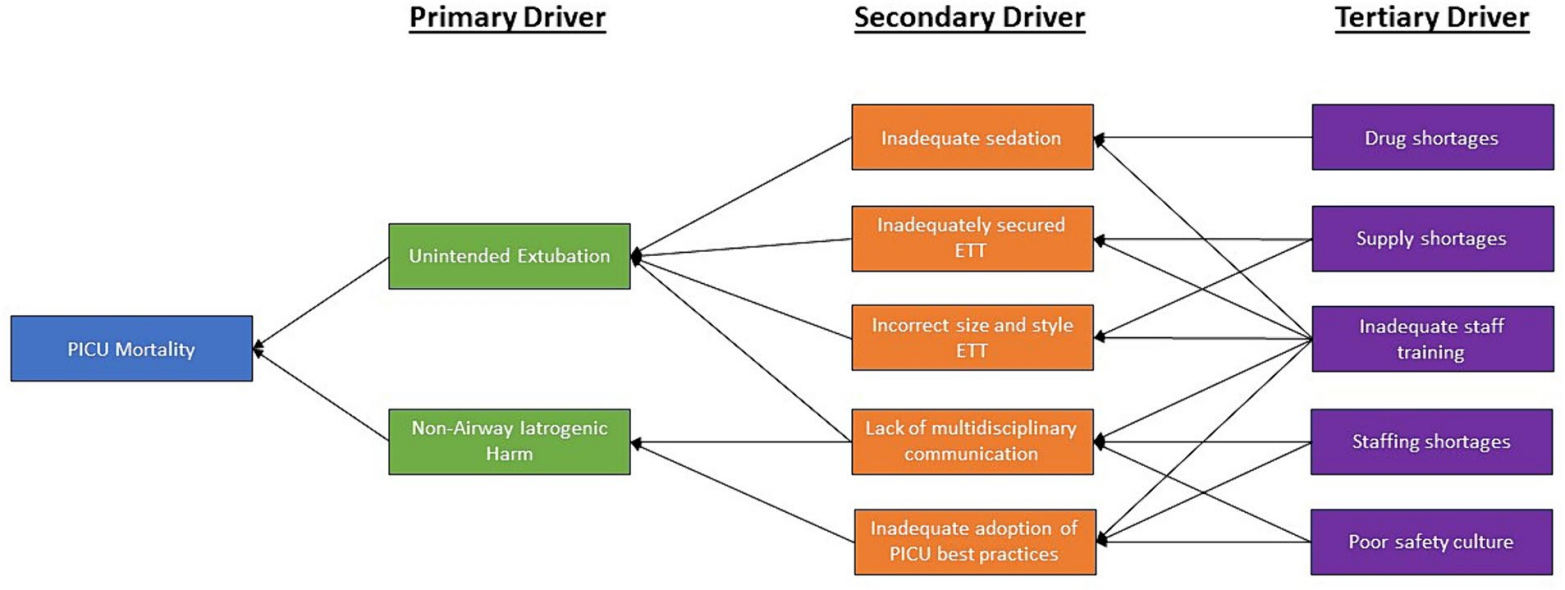
Careways Impact Pathway Model. The Careways Impact Pathway Model elucidates the pathways through which the planned Quality Improvements intervention attains its specified objectives, delineates the causal mechanisms by which the intervention operates, as well as demonstrates the interconnectedness and mutual reinforcement among its constituent elements. Within this framework, balancing factors are elements that might counteract or moderate the effects of the intervention, helping to maintain equilibrium within the system.

Driver diagrams are designed to elucidate the logical links between aspects of the environment, culture, and workflows of an institution with the outcomes that a team is trying to change in a QI project. Primary drivers are directly linked to the overall outcome, while secondary drivers primarily influence these primary drivers. Tertiary drivers have a more direct relationship with secondary drivers, and so forth. Understanding drivers provides targets for interventional strategies to improve the outcome of interest. We designed a driver diagram to describe pediatric mortality in the PICU through discussion with a multidisciplinary team of OA members and local clinicians (Figure 3). The model was iteratively refined with the assistance of a QI expert until the team felt that consensus was reached.

### Impact Pathway Model

**Figure 4:**
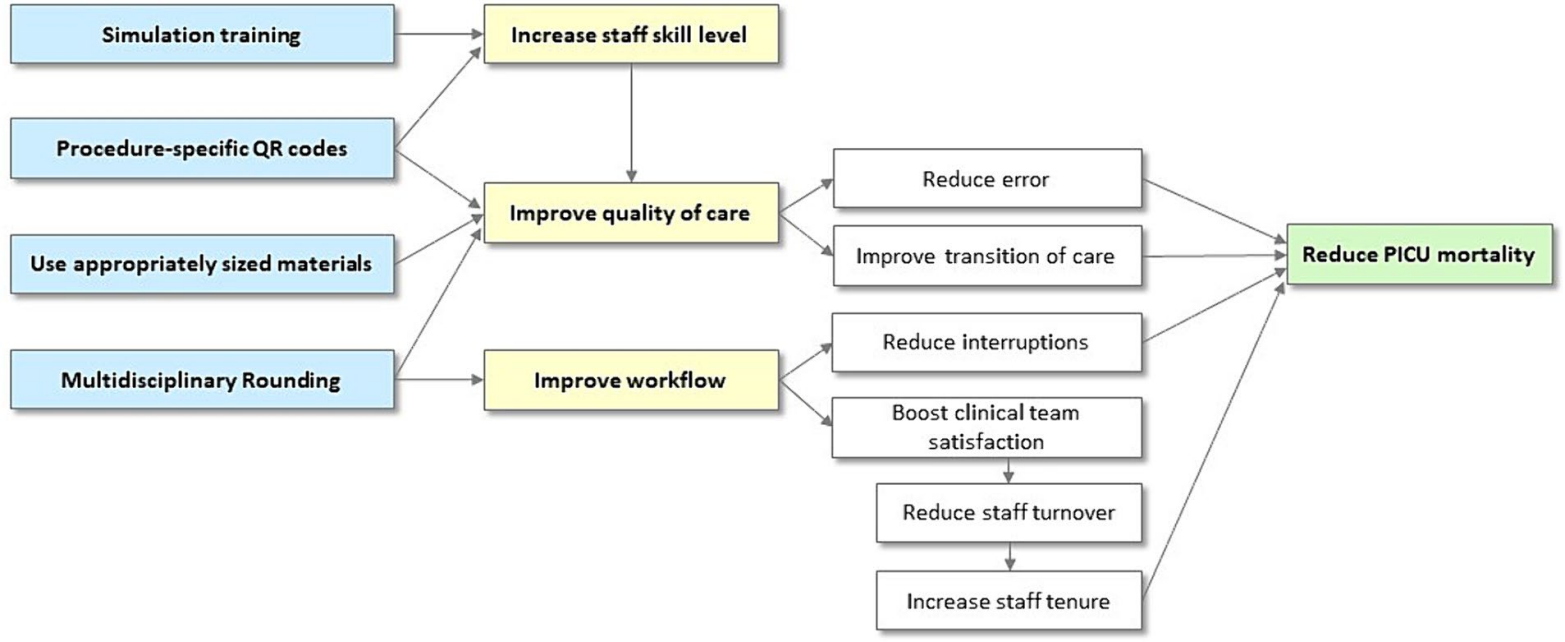
Impact Pathway Model used to comprehend and monitor changes to the causal pathway that we believe will lead to the desired outcomes.

Driver diagrams and Impact Pathways are closely related as they both serve to delineate the causal relationships between interventions, intermediate outcomes, and ultimate goals. They can both serve to suggest process metrics and future data collection strategies for understanding the exact impact of an intervention. Driver diagrams focus more broadly on describing the system, which can be useful for considering targets for interventions, while impact pathways can help clearly describe how an intervention is impacting the outcome of interest. Using the same methods as for the driver diagram, we developed an Impact Pathway Model (Figure 4) to comprehend and monitor changes to the causal pathway that we believe will lead to the desired outcomes. We began with the four components of the intervention and sought as a group to connect those to the outcome of interest.

## Analysis and findings

### Data

An analytic data file for PICU patients, under 18 years of age, undergoing mechanical ventilation was constructed. The data file includes binary variables for gender, intubation status, accidental intubation, and mortality. The dataset also includes date of intubation, age, and weight, PRISM severity of illness scores, and diagnosis. Data were cleaned to remove duplicate entries, diagnosis codes were consolidated into 4 collapsed categories for improved clarity, and binary variables (e.g., accidental extubation) with values greater than 1 were set to 1, e.g., multiple AEs were reset to binary yes/no was the member AE. Missing data was treated as missing, i.e., not imputed.

147 patients met eligibility criteria in Epoch 3. There was no missing outcome data. 45·5% were female. The mean age was 3·2 years and the mean weight was 18·16 kg. The most common diagnosis was sepsis (38·9%) followed by pneumonia (35·8%) and trauma (7·0%). Epoch 2 had 98 patients, and Epoch 3 had 147 patients. The groups were similar regarding age, sex, weight, severity of illness (PRISM scores), and diagnosis (Table 2). The Pediatric Risk of Mortality (PRISM) score was developed from the Physiologic Stability Index (PSI) to reduce the number of physiologic variables required for pediatric ICU (PICU) mortality risk assessment and to obtain an objective weighting of the remaining variables (12, 13).

**Table 2:** Demographics.

	Epoch 2 (*N* = 98)	Epoch 3 (*N* = 147)	*p* value
Female sex – *n* (%)	55 (45·9)	57 (45·5)	0.22
Age in years – mean (SD)	2·9 (3·99)	3·2 (3·2)	0.55
Weight in kg – mean (SD)	14·13 (16·83)	18·16 (15·64)	0.06
Diagnosis			
Sepsis – *n* (%)	39 (39·8)	58 (38·9)	0.24
Pneumonia – *n* (%)	35 (35·6)	32 (35·8)	
Trauma – *n* (%)	9 (9·2)	37 (7·0)	
Other – *n* (%)*	15 (15·3)	20 (18·3)	
PRISM Severity Score (SD)	16.61 (8.08)	16.59 (6.95)	0.06

Comparison of patient demographics between the immediate post-intervention cohort and the long-term post-intervention cohort indicate no difference in sex or weight, though patients in the long-term post-intervention cohort were slightly older with more trauma and “other” diagnoses than in the short-term post-intervention cohort. There were more than ten different diagnostic possibilities in the long-term post-intervention cohort such that the top three are listed followed by “other” which includes meningitis, Guillain-Barré, metabolic, cardiac, seizures, and other infections. Diagnosis did not significantly impact mortality either when utilizing the “other” category to describe the remaining seven diagnoses or when all categories were evaluated by logistic regression analysis. ^*^Other diagnoses include meningitis, Guillain-Barré, metabolic, cardiac and seizures. ^**^Epoch 1 data other than PRISM severity scores have been reported previously. The PRISM severity score for Epoch1 was 16.35 and was neither significantly different that the PRISM score for Epoch 2 or 3.

Given the hypothesis that PICU mortality in low/middle resources countries is unusually high relative to higher resourced settings due to avoidable errors, our group felt that a 15% or greater diminishment of all cause child mortality following our education and culture change intervention was achievable and would be clinically meaningful. Assuming a Power of 0·8 and alpha of 0·05, we needed 87 children in Epoch 3. The final sample included 98 patients from Epoch 2 and 147 patients in Epoch 3.

### Analysis

The primary outcome was all cause mortality in intubated PICU patients in Epoch 2 compared to Epoch 3, to assess for sustainment of the improvements noted in the earlier study. Secondary analyses consisted of comparisons between proportions of unexpected events/100 days mechanical ventilation. This was assessed using an independent *t*-tests for differences in means, 2-sample test for equality of proportions with continuity correction, and linear regression to account for covariates, including age, sex, PRISM severity of illness scores, and admitting diagnosis. As an exploratory measure, we also report the list of sustained QI practices and new practice changes observed in Epoch 3.

Comparing Epoch 2 to Epoch 3, the overall PICU mortality decreased from 22.4 (std 0.42) to 9.5% (std 0.29): *p* value: 0.002 (confidence intervals: 0.05;0.21). The observed significant decrease in mortality persisted even after controlling for age, weight, PRISM severity of illness scores, and initial diagnoses. The regression model yielded an adjusted R-squared of 0.046, indicating that approximately 4.6% of the variance in mortality was explained by the predictors (F-statistic: 3.198; *p* = 0.008).

### Secondary outcomes

Comparing Epoch 2 to Epoch 3, the unexpected events per 100 days of mechanical ventilation did not significant change 3.52 to 3.30; *p* value: 0.29 (confidence intervals: −0.10;0.30) (2-sample test for equality of proportions with continuity correction).

### Qualitative observations

Observational data was notable for sustainment of the QI practices implemented prior to Epoch 2 and additional locally driven QI measures. The modified pragmatic QI program the local team developed to promote and ensure communication and multi-disciplinary care planning was utilized in all 147 patients seen in the PICU during this time period. Table 1 details the additional unanticipated practice changes implemented by the local health care team.

### System modeling

The driver diagram and impact map are shown in Figures 3, 4, respectively. In the impact diagram, each component contributes to, but is not uniquely responsible for, reducing interruptions, reducing errors, reducing PICU staff turnover, and thereby reducing PICU mortality. Both of these diagrams can provide potential process metrics to validate the underlying logic linking the interventions to the outcomes. The driver diagram can additionally provide potential targets for additional interventions to further improve PICU mortality.

## Discussion

We observed sustained local improvement in this QI project with continued decreases in PICU mortality in intubated pediatric patients 36 months following implementation of an educational and QI intervention aimed at increasing multidisciplinary teamwork and decreasing airway related morbidity and mortality. Interestingly, the qualitative observations showed a significant number of locally initiated QI projects in addition to our initial QI intervention. We believe these changes indicate a burgeoning PICU safety culture and may help to explain why the decrease in mortality from Epoch 1 to Epoch 2 was not only sustained but continued to improve in Epoch 3 from 22·4% to 9·5%.

It is of interest that secondary measures/ adverse events such as UEs changed significantly between Epochs 1 and 2 (9), but not between Epochs 2 and 3, whereas mortality continued to significantly drop between Epochs 1, 2, and 3. It may be that AE drops immediately to the lowest possible level (or lowest reasonable level without added intervention) and remained stable thereafter, and that subsequent mortality decreases were due to (a) other components of the intervention bundle, (b) changes in the severity of cases, (c) exogenous effects, or (d) a combination of these three. Going forward, systematic collection of data related to elements in the Impact Pathway will enable us to tease apart these connections and form a more refined understanding of the causal connections.

Many global health studies have demonstrated short-term favorable impacts on patient care immediately following implementation of QI processes in low-resource settings (14, 15), and some have reported on long-term sustainability of process improvement, retention of provider skills and strengthening of partnerships between teams in high and low-resource settings (16, 17). Yet few global health investigators have demonstrated long-term effectiveness of QI programs in LMIC on patient outcomes, often citing lack of robust data systems to reliably measure morbidity and mortality and attrition of provider skills without ongoing training (18, 19). While we acknowledge that our intervention alone may not account for the sustained improvement observed in this study, we believe that our Driver Map and Impact Pathway models, combined with forthcoming additional data collection and a comprehensive analysis will address significant gaps in understanding the relationships and long-term impacts of these programs, contributing to the broader context of global health studies demonstrating both short-term improvements and long-term sustainability in low-resource settings.

### Study strengths and limitations of the analysis

One key strength of our analysis was the use of a reliable database to accurately measure the impact of our intervention on patient outcomes (18, 19). Our research group attempted to account for possible confounders that might influence our findings related to a decrease in child mortality. Data was collected for a similar amount of time and during a similar season of the year to account for possible seasonal variation of disease in EL Salvador. Choosing a similar season was not planned in advance (because no one foresaw covid) but was considered while planning post covid trips as a deliberate choice made to help address/control for exogenous seasonal/country-level effects. Age, sex, PRISM severity of illness indices, and diagnosis were identified as possible factors underlying severity of illness and accounted for (age and diagnosis were specifically accounted for by regression analysis post-hoc as there were imbalances in these seen in Table 2).

We make no claims that our intervention alone led to the sustained, and continued, improvement observed in this study. Rather, our observational data and proposed Impact Pathway model suggest that the improvement is multifactorial and complex. Future research is underway to collect quantitative data to validate the Impact Pathway model and understand the impact of the various QI programs now at this site. This work will increase our understanding of the system to promote future improvement.

### Future directions

The low-cost nature of our initial intervention and its sustainable impact with only light-touch, internet-based support suggests the opportunity for scalability in the future. We aim to roll out this intervention in three additional PICUs in other LMIC countries in Central and South America to better understand how this intervention is locally adapted, what needs to be taken into account for future scalability of the intervention, and whether this impressive improvement in mortality can be recreated elsewhere. This future work will generate additional data which will be used to refine and validate the Impact Pathway model, furthering our understanding of how improvements occur in LMIC PICUs. Although a direct causal relationship between the adoption of this QI improvement program and reduction in child mortality may be difficult to prove, we hope to see a mirroring of this significant decrease in child mortality in these next three countries’ PICUs post-intervention as well. Our chief goal will be to work further to optimize these forms of sustainable quality improvement programs focusing on enhanced communication and multidisciplinary care.

## Conclusion

Overall, we present data showing a sustained improvement in reduction of mortality in intubated PICU patients following a low-cost, low-touch educational and QI intervention aimed at improving multidisciplinary care and uptake of clinical best practices. A direct causal relationship between the implementation of this intervention and reduction in child mortality remains difficult to prove. However, we propose an Impact Pathway model positing potential causal pathways based on our observations of the intervention implementation and the addition of other QI interventions undertaken by the local team following the initial intervention. Work is underway to enhance the implementation of the intervention across a wider range of settings and gather comprehensive data on its functional components and their association with patient outcomes. We eagerly anticipate advancing this work to cultivate a replicable and sustainable model for Quality Improvement programs across low-resource countries, transcending the confines of a PICU and extending to operating rooms, wards, and every facet of a child’s journey from home to hospital and back home again.

## Data availability statement

The raw data supporting the conclusions of this article will be made available by the authors, without undue reservation.

## Ethics statement

The studies involving humans were exempted for consideration as research by the Massachusetts General Hospital’s Institutional Review Board as they were considered Quality Improvement studies. The studies were conducted in accordance with the local legislation and institutional requirements and were formally approved by the Benjamin Bloom Hospital Institutional Review Board, the local hospital where the work was being done. The ethics committee/institutional review board waived the requirement of written informed consent for participation from the participants or the participants’ legal guardians/next of kin because this was aggregate data and no PHI was recorded and the study was a QI study.

## Author contributions

PY: Writing – review & editing, Writing – original draft. KC: Writing – review & editing, Writing – original draft. AS-W: Writing – review & editing, Writing – original draft. JB: Writing – review & editing, Writing – original draft. LF: Writing – review & editing, Writing – original draft. SH: Writing – review & editing, Writing – original draft. AR: Writing – review & editing, Writing – original draft. AC: Writing – review & editing, Writing – original draft, Project administration. AN: Writing – review & editing, Writing – original draft. AJ: Writing – review & editing, Writing – original draft. EZ: Writing – review & editing, Writing – original draft. CH: Writing – review & editing, Writing – original draft.
